# The relationship between diastolic blood pressure and coronary artery calcification is dependent on single nucleotide polymorphisms on chromosome 9p21.3

**DOI:** 10.1186/s12881-014-0089-2

**Published:** 2014-09-04

**Authors:** Daniel S Kim, Jennifer A Smith, Lawrence F Bielak, Chun-Yi Wu, Yan V Sun, Patrick F Sheedy, Stephen T Turner, Patricia A Peyser, Sharon LR Kardia

**Affiliations:** 1 Department of Epidemiology, University of Michigan School of Public Health, 1415 Washington Heights, Ann Arbor 48109, MI, USA; 2 Department of Genome Sciences, University of Washington School of Medicine, Seattle, WA, USA; 3 Division of Medical Genetics, Department of Medicine, University of Washington School of Medicine, Seattle, WA, USA; 4 Department of Epidemiology, Emory University School of Public Health, Atlanta, GA, USA; 5 Department of Diagnostic Radiology, Mayo Clinic, Rochester, MN, USA; 6 Division of Nephrology and Hypertension, Department of Internal Medicine, Mayo Clinic, Rochester, MN, USA

**Keywords:** Epidemiology, Genetics of cardiovascular disease, Atherosclerosis risk factors, Other arteriosclerosis

## Abstract

**Background:**

Single nucleotide polymorphisms (SNPs) within the 9p21.3 genomic region have been consistently associated with coronary heart disease (CHD), myocardial infarction, and quantity of coronary artery calcification (CAC), a marker of subclinical atherosclerosis. Prior studies have established an association between blood pressure measures and CAC. To examine mechanisms by which the 9p21.3 genomic region may influence CHD risk, we investigated whether SNPs in 9p21.3 modified associations between blood pressure and CAC quantity.

**Methods:**

As part of the Genetic Epidemiology Network of Arteriopathy (GENOA) Study, 974 participants underwent non-invasive computed tomography (CT) to measure CAC quantity. Linear mixed effects models were used to investigate whether seven SNPs in the 9p21.3 region modified the association between blood pressure levels and CAC quantity. Four SNPs of at least marginal significance in GENOA for a SNP-by-diastolic blood pressure (DBP) interaction were then tested for replication in the Framingham Heart Study’s Offspring Cohort (N = 1,140).

**Results:**

We found replicated evidence that one SNP, rs2069416, in *CDKN2B-AS1*, significantly modified the association between DBP and CAC quantity (combined P = 0.0065; Bonferroni-corrected combined P = 0.0455).

**Conclusions:**

Our results represent a novel finding that the relationship between DBP and CAC is dependent on genetic variation in the 9p21.3 region. Thus, variation in 9p21.3 may not only be an independent genetic risk factor for CHD, but also may modify the association between DBP levels and the extent of subclinical coronary atherosclerosis.

## Background

Coronary heart disease (CHD) is the leading cause of morbidity and mortality in the United States and other Western countries, and accounts for approximately one-third of all deaths in adults over age 35 [1]. The vast majority of CHD cases arise from pathologic processes (usually atherosclerotic plaque) in the coronary arteries. The extent of atherosclerosis in the coronary arteries can be assessed non-invasively by measurement of coronary artery calcification (CAC) quantity via computed tomography (CT) [2]. CAC quantity is heritable [3], correlates with increased burden of atherosclerotic plaque in the coronary arteries [4], and is a predictor of incident CHD in multiple ethnic populations after adjustment for established risk factors (RFs) [2],[5], such has age, sex, cigarette smoking [6], hypertension [7], hyperlipidemia [8], and diabetes [9].

A region on chromosome 9p21.3, within the *CDKN2B* anti-sense RNA (*CDKN2B-AS1*), nearby the *CDKN2A* and *CDKN2B* genes, has been found to be strongly associated with CHD and myocardial infarction [10]-[14]. Single nucleotide polymorphisms (SNPs) in this region also have been associated with CAC, a subclinical measure of CHD [15]. Little is known about the underlying mechanisms of action. To date, no studies have considered whether variations in this genomic region modify the association between blood pressure and CAC quantity.

The Genetic Epidemiology Network of Arteriopathy (GENOA) and the Framingham Heart Study (FHS) participated in the Cohorts for Heart and Aging Research in Genetic Epidemiology (CHARGE) effort to identify genetic regions for CAC quantity [15] using a genome wide association study (GWAS). GENOA is a study of sibships and is unique among CHARGE cohorts in that participants were ascertained based on a history of hypertension in their sibship and thus are at high risk for sequelae of hypertension [16]. Interestingly, there were no significant associations between SNPs in the 9p21.3 region and CAC in GENOA that were found in the other cohorts in CHARGE [15]. The background of a strong personal and/or family history of early onset hypertension, a risk factor for CAC, may have contributed, in part, to the differences at 9p21.3 for GENOA compared to other cohorts. Given GENOA’s study design, we investigated whether there was any evidence of SNP-blood pressure interactions on CAC quantity in GENOA that replicated in the Framingham Heart Study (FHS) after consideration of other established RFs, to better understand the potential influence of genetic variation in the 9p21.3 region on the pathogenesis of CHD.

## Methods

### Subjects

Both the GENOA and FHS cohorts were comprised of European Americans, who provided written informed consent. The use of GENOA data and dbGaP FHS data for the purpose of this study was approved by the University of Michigan Health Sciences and Behavioral Sciences Institutional Review Board.

The GENOA study is a longitudinal community-based study of sibships to identify genes influencing blood pressure (BP) and its target organ damage sequelae [16],[17]. Sibships with at least two adults with clinically diagnosed essential hypertension before age 60 were recruited. All other members of the sibship were invited to participate regardless of their hypertension status. During the first exam (1995–2000), 1,583 individuals were examined in the Rochester, MN field center. During the second exam (2000–2004), 1,241 participants were re-examined to measure RFs, including systolic blood pressure (SBP), diastolic blood pressure (DBP), and CAC quantity. Exclusion criteria were secondary hypertension, alcoholism, drug abuse, pregnancy, or active malignancy. Of these 1,241 participants, 974 had RFs, genotypes, and CAC measures and comprised the GENOA discovery cohort. The GENOA participants were from 435 sibships, with an average size of approximately 2.25 siblings per sibship.

The FHS was initiated in 1948 by systemically enrolling two-thirds of the households in Framingham, MA. In 1972, the Framingham Offspring cohort study was initiated, and included 5,124 offspring of the original cohort and offspring spouses [18]. Utilizing the dbGaP mechanism (http://www.ncbi.nlm.nih.gov/gap) to request data, we downloaded version 3 data of the FHS genotype and phenotype files. The replication sample for this study was comprised of 1,140 FHS Offspring cohort members who attended a baseline clinic visit in 1998–2001 (exam 7), underwent a CT examination an average of 4 years later (2002–2005), and had available genotype data. FHS participants were from 450 sibships, with an average sibship size of approximately 2.53 siblings.

### Risk factor measures

Detailed information on measurement of RFs is provided in the Additional file 1: Materials.

### CAC measurement

CAC was measured in GENOA participants with an Imatron C-150 electron beam CT (EBCT) scanner (Imatron Inc., South San Francisco, CA) using a previously described protocol [19] to assess CAC quantity using dual scan runs. FHS utilized an 8-slice Multi Detector CT (MDCT) scanner (Lightspeed Ultra; General Electric Medical Systems, Milwaukee, WI) to measure CAC quantity [20]. CAC was defined as hyperattenuating foci in a coronary artery that was at least 1.0 mm^2^ in size, with a radiograph attenuation coefficient (CT number) above 130 Hounsfield Units throughout the focus. Total CAC score in the heart was quantified by summing the CAC scores across the four main epicardial arteries using the Agatston method [21]. Detectable CAC was defined as a CAC score at least 1.0. In GENOA, the average CAC score of two sequential CAC scans was used for all analyses. The CAC score was natural log-transformed (ln(CAC score + 1)) to reduce skewness. Prior studies have established a significant correlation between CAC measures from EBCT and MDCT scanners [22],[23].

#### Genotyping/Imputation

In the GENOA Study, SNPs were measured using the Affymetrix Genome-Wide Human SNP Array 6.0 platform (Santa Clara, CA). In the FHS, SNPs were measured using the Affymetrix Human Mapping 500 K Array Set and 50 K Human Gene Focused Panel (Santa Clara, CA). Participants were excluded if they had an overall SNP call rate < 95% or sex mismatch between genotypic and phenotypic measurement. SNPs were excluded if they had unknown chromosomal location, a call rate < 95% or a minor allele frequency (MAF) less than 0.01. From the remaining SNPs, imputation was performed using data from HapMap release 22, build 36, CEU population via MACH version 1.0.16 (http://www.sph.umich.edu/csg/abecasis/MaCH). Only SNPs with an imputation quality (RSQ) ≥ 0.8, MAF > 0.01, and Hardy Weinberg Equilibrium (HWE) P-Value > 1.0 × 10^−6^ were included in the analyses.

In the CHARGE CAC GWAS, 75 imputed SNPs in the 9p21.3 region had a p-value < 10^−6^ for association with CAC quantity [15]. Due to the high degree of linkage disequilibrium (LD) between the SNPs, we reduced the number of SNPs in the analysis by utilizing LDSelect, which created seven tagSNPs based on an LD threshold of 0.60 [24]. The seven SNPs studied and their pairwise LD r^2^ are presented in Additional file 1: Figure S1.

### Statistical Analysis

Means and standard deviations were computed for continuous variables, and percentages were calculated for discrete variables. Linear mixed effects models with family as a random intercept were used to examine differences in characteristics between GENOA and Framingham participants. To examine the associations between RFs and CAC score in each cohort, a linear mixed effects model was fit with ln(CAC score + 1) as the outcome and the following RFs as covariates: age, sex, SBP, DBP, anti-hypertensive medication use, diabetes status, fasting glucose levels, ln(pack years + 1), the ratio of low density lipoprotein cholesterol to high density lipoprotein cholesterol (LDL:HDL), and statin drug use. Residuals from this model were then used as the outcome variable in linear mixed effects models to examine the association between each of the 9p21.3 SNPs and CAC score. Linear mixed effects modeling with family as a random intercept was used to properly account for sibship structure among the participants in each cohort while retaining a valid type I error rate [25].

SNP-by-BP interactions were investigated by first obtaining the residuals from a linear mixed effects model that included all the covariates except the specific BP measure of interest. Linear mixed effects models were used to assess whether a SNP-by-BP measure interaction predicted the residuals adjusting for the SNP and the BP measure of interest. For all analyses, the residual of ln(CAC score +1) from regression of selected RFs was the outcome variable. From this point onward, the term “CAC quantity” is used to refer to residual ln(CAC score + 1).

Imputed SNP genotype dosages were used in an additive genetic model for all analyses. SNPs with marginally significant (P ≤ 0.10) interaction terms in the GENOA cohort were tested in the FHS cohort.

Statistical significance was defined as having P ≤ 0.05 in both GENOA and FHS and the coefficient for interaction being different from zero. All tests were two-sided. Combined P-Value Tests were performed using MetaP (http://compute1.lsrc.duke.edu/softwares/MetaP/metap.php) and the reported statistic reflects Stouffer’s z, which considers both P-values and sample sizes [26]. Combined P-values were corrected using the Bonferroni method in order to account for testing seven SNPs. Replication was declared when P-values for the interaction term were at least marginally significant in both cohorts and the effects for the SNP, BP measure, and interaction terms were consistent between cohorts based on genotype-specific plots of the relationship between BP and CAC quantity.

**Figure 1:**
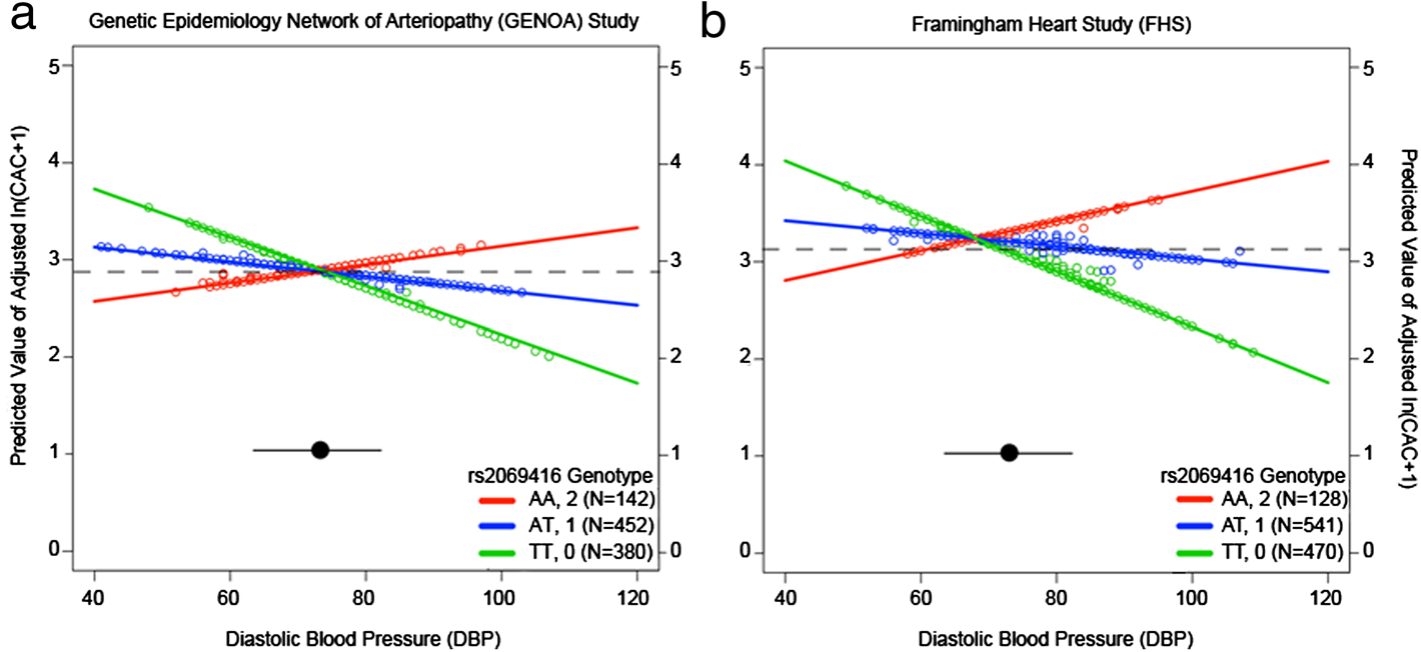
**rs2069416 genotype dependent interaction with diastolic blood pressure (DBP, mmHg) on estimated ln(CAC + 1) adjusted for age, sex, systolic blood pressure, anti-hypertensive medication use, diabetes status, fasting glucose levels, ln(pack years + 1), LDL:HDL, and statin drug use. Panel (a)** shows the rs2069416 genotype-specific relationship between DBP and the estimated adjusted ln(CAC + 1) for GENOA participants. **Panel (b)** shows the rs2069416 genotype-specific relationship between DBP and the estimated adjusted ln(CAC score + 1) for FHS participants. Dashed lines represent the mean value of ln(CAC score + 1) for each cohort. The black circle and accompanying line represent the mean and standard deviation, respectively, for DBP. Colored circles represent the predicted value of ln(CAC score + 1) for each study participant, according to their genotype.

Statistical analyses were conducted using the R statistical language (http://www.r-project.org/). The statistical methodology for the creation of the LD plot in Additional file 1: Figure S1 and the plotting of the predicted values of adjusted CAC quantity presented in Figure 1 are described in Additional file 1: Materials.

## Results

Descriptive characteristics for the GENOA and FHS cohorts are presented in Table 1. The percentage of participants with detectable CAC, CAC score > 100, and CAC score >300 was 68.6%, 31.2%, and 17.1%, respectively, in GENOA and 68.39%, 37.1%, and 21.1%, respectively, in FHS (data not shown). Significantly more GENOA than FHS participants were women (59.0% vs. 54.3%, respectively; P = 0.03). GENOA participants had significantly lower mean age, SBP, and ln(pack years + 1) than FHS participants. GENOA participants had a significantly higher prevalence of hypertension, anti-hypertensive medication use, diabetes, and statin use but lower past or active tobacco use than FHS participants. The association between RFs and CAC quantity are presented in Additional file 1: Table S1. As expected, many but not all RFs were associated with CAC quantity in each cohort.

**Table 1:** Baseline characteristics of GENOA and FHS

	**GENOA**	**FHS**	**P**^**‡**^
	**N = 974**	**N = 1,140**	
Age, years	58.41 (±10.17)	59.28 (±8.98)	0.67
Women, %	59.02%	54.35%	0.03
Participants with detectable CAC, %	68.60%	68.39%	0.92
ln(CAC score + 1)	2.88 (±2.56)	3.15 (±2.66)	0.16
Hypertension, %	72.17%	37.84%	<0.0001
Anti-hypertensive medication use, %	67.58%	27.83%	<0.0001
Systolic blood pressure, mmHg	131.20 (±16.87)	125.04 (±17.59)	<0.0001
Diastolic blood pressure, mmHg	74.26 (±9.16)	74.56 (±9.37)	0.60
Diabetes, %	13.96%	7.46%	<0.0001
Fasting glucose, mg/dL*	97.27 (±10.71)	96.66 (±9.78)	0.43
Past or Active tobacco use, %	45.26%	56.19%	0.0001
ln(Pack years + 1)	1.28 (±1.58)	2.56 (±1.01)	0.03
Statin Use, % †	24.57%	15.59%	<0.0001
LDL:HDL ratio	2.55 (±1.04)	2.56 (±1.01)	0.71

GENOA = Genetic Epidemiology Network of Arteriopathy; FHS = Framingham Heart Study Offspring cohort; CAC = coronary artery calcification; HDL = high density lipoprotein; LDL = low density lipoprotein; ln = natural logarithm; Statin = HMG-CoA Reductase Inhibitor.

*Fasting glucose mean and standard deviation calculated in subset of non-diabetics (GENOA: N = 844, FHS: N = 1054).

†Statin use was missing for 5 FHS participants.

^**‡**^P-value from a linear mixed effects model to test the difference between the two cohorts’ means (quantitative traits) or the difference between the two cohorts’ frequencies (qualitative traits), accounting for sibship structure.

The pairwise LD relationships between the seven SNPs studied in the 9p21.3 region are presented in Additional file 1: Figure S1. The associations between the seven SNPs and CAC quantity are presented in Additional file 1: Table S2. In GENOA, only rs3731239, a *CDKN2A* intronic SNP, was significantly associated with adjusted CAC quantity. In contrast, three SNPs within or nearby *CDKN2B-AS1* (rs1333049, rs1333050, and rs1333040), were significantly associated with adjusted CAC quantity in FHS.

Of the seven SNP-by-DBP interactions tested, four were at least marginally significant in the GENOA discovery cohort (rs2069416, rs1333040, rs1333049, and rs1333050) and tested for replication in FHS (Table 2). Only the rs2069416-by-DBP interaction was significant in both cohorts (GENOA: P = 0.04; FHS: P = 0.033; combined P = 6.50 × 10^−3^). This interaction remained significant after Bonferroni correction (Bonferroni-corrected combined P = 0.0455). Although rs1333040 and rs1333049 were not at least marginally significant for a SNP-by-DBP interaction term in FHS, in the combined analyses using Stouffer’s z-test, the SNP-by-DBP interaction term had P < 0.05 for each SNP (rs1333040 combined P = 0.0246; rs1333049 combined P = 0.0291). However, neither of these interactions were significant after Bonferroni correction (rs1333040 Bonferroni-corrected combined P = 0.1722; rs1333049 Bonferroni-corrected combined P = 0.2037). No SNP-by-SBP interaction terms were at least marginally significant in GENOA, and therefore none were tested for replication in FHS.

**Table 2:** SNP-by-DBP interactions on CAC quantity*

**SNP**	**Closest reference gene**^**†**^	**Coded allele**	**GENOA CAF**	**FHS CAF**	**GENOA interaction P**	**FHS Interaction P**	**Combined P**^**‡**^	**Bonferroni-corrected combined P**^**§**^
rs2069416	*CDKN2B-AS1*	A	0.3666	0.3351	0.047	0.033	6.50 × 10^−3^	0.0455
rs1333040	*CDKN2B-AS1*	C	0.5829	0.6031	0.012	0.250	0.0246	0.1722
rs1333049	*(CDKN2B-AS1)*	C	0.5107	0.5089	0.014	0.273	0.0291	0.2037
rs1333050	*(CDKN2B-AS1)*	C	0.6652	0.7009	0.069	0.565	0.2008	1
rs2069418	*CDKN2B-AS1*	C	0.5447	-	0.289	-	-	-
rs3218009	*CDKN2B-AS1*	C	0.8106	-	0.370	-	-	-
rs3731239	*CDKN2A*	C	0.3766	-	0.711	-	-	-

SNP = single nucleotide polymorphism; DBP = diastolic blood pressure; CAF = coded allele frequency; GENOA = Genetic Epidemiology Network of Arteriopathy; FHS = Framingham Heart Study Offspring cohort.

*Adjusted for age, sex, systolic blood pressure, anti-hypertensive medication use, diabetes status, fasting glucose levels, ln(pack years + 1), LDL:HDL ratio, and statin drug use.

†Intergenic SNPs are represented in parentheses naming the nearest gene, e.g. *(CDKN2B-AS1).*

^**‡**^Combined P was achieved through Stouffer’s z-test, which considers both p-values and sample sizes.

^§^Bonferroni-corrected for testing 7 SNPs.

Rs2069416-by-DBP interaction plots are presented in Figure 1a and 1b for GENOA and FHS, respectively. The interaction plots demonstrate a decrease in predicted CAC quantity with increasing DBP for participants with one or two copies of the T allele in each cohort.

## Discussion

The current, clinically applicable understanding of the pathogenesis of CHD is generally limited to RF exposures and basic genetics in the form of the family history. Yet this base of knowledge alone does not begin to explain the complex etiology of CHD. Recent GWAS have identified more than 30 genetic loci associated with CHD [10],[11] and other loci (both overlapping and novel) associated with CAC quantity [15]. However, these identified variants have small effect sizes, which have raised the question of what accounts for the missing heritability of CHD. There are multiple theories to account for the missing heritability of CHD. These include, but are not limited to, unscreened control data [27], biased sample collection [28], non-additive genetic effects, and that unidentified variants, rare and common, account for a majority of the risk for CHD and other common diseases [29]. These theories, though, do not account for the possibility of gene-by-environment interactions and how they could contribute to missing heritability for traits such as CAC quantity. Accordingly, we have demonstrated a novel, replicated finding for a SNP in the 9p21.3 region: that a specific common variant on chromosome 9 provides a context for gene-by-BP interactions, thus defining the range of influence that blood pressure can have in atherogenesis and plaque deposition.

Specifically, we have extended accumulated empirical evidence regarding the 9p21.3 region and CHD and present significant and replicated evidence that rs2069416 interacts with DBP to exacerbate the extent of CAC in a genotype dependent manner. While in the CAC GWAS, that ignored any interactions, GENOA and FHS had discordant results for the 9p21.3 region, once the interaction with DBP was considered, inferences for a specific SNP became concordant. Moreover, no SNPs considered were significant for primary association with CAC in both cohorts; it was only in the context of a SNP-by-DBP interaction that the association with CAC was identified and replicated.

The lack of evidence for significant SNP-by-SBP interactions in the GENOA discovery cohort may reflect the high (67.6%) use of anti-hypertensive medications that specifically lower SBP, while largely leaving DBP levels unaffected. Moreover, our findings of CAC quantity being higher with lower DBP likely reflects pulse pressure due to the inclusion of both SBP and DBP (and the control for one measure of BP when the other is being tested for interaction). Pulse pressure has been demonstrated to increase with age due to increases in SBP and decreases in DBP [30]. While tests for interactions between SNPs in 9p21.3 and pulse pressure were not significant in GENOA (data not shown), there remains the possibility that pulse pressure is contributing to the observations in this study. There is a complex relationship between pulse pressure and quantity of CAC: at younger ages (<50 years of age), SBP and DBP have been shown to be positively associated with quantity of CAC and likely act as surrogates of arterial resistance; however, quantity of CAC is more closely associated with pulse pressure in subjects older than 50 years, which likely reflects large-artery stiffness [31]. Similar age-related effects of pulse pressure have also been reported in the FHS, with an increase in pulse pressure after 50–60 years of age [30] and pulse pressure becoming the strongest predictor of CHD risk after 59 years of age in FHS [32]. In GENOA, as pulse pressure increased, CAC quantity also increased, regardless of genotype at rs2069416 (Additional file 1: Figure S2). However, both cohorts included participants younger than age 50 and older than age 50. Moreover, there was not sufficient power to consider a SNP-by-pulse pressure interaction in age specific strata or to consider three-way interactions that included age, SNP and pulse pressure.

A recent large GWAS for blood pressure identified 28 loci that, to the best of our knowledge, do not overlap with GWAS loci identified for CAC quantity or CHD [33]. Our novel finding of a replicated interaction between a 9p21.3 SNP and DBP suggests a new genetic pathway through which BP variation contributes to variation in CAC quantity and thereby the potential pathogenesis of CHD. Specifically, it suggests that the *CDKN2B-AS1* region, which is critical for numerous atherosclerotic phenotypes [15],[34],[35], but not associated with blood pressure phenotypes [33], may confer genetic risk through differential atherosclerotic plaque development for a given level of blood pressure. This mechanism may also be shared with other genomic regions associated with CHD and other atherosclerotic phenotypes, but not with blood pressure.

Despite findings of a replicated SNP-by-DBP interaction in the 9p21.3 region, knowledge is still lacking about the specific mechanisms by which genetic variants in the 9p21.3 region contribute to CAC extent and subsequent CHD pathogenesis. The 9p21.3 region has previously been consistently associated with CHD and related phenotypes, such as familial, premature CAD [36], abdominal aortic aneurysms [14], vascular wall stiffness [37], increased platelet reactivity [34], subclinical carotid artery disease [38], and ankle-brachial index [39]. The implicated SNP within the 9p21.3 region is near protein-coding genes and also overlaps with an antisense non-coding RNA (also known as *ANRIL* or DQ485453) [40]. Our top SNP-by-DBP interaction occurred with a non-coding variant, rs2069416, which is not in LD with any coding or obvious regulatory variants. Additionally, Visel et al. found that deletion of the mouse-analogue of the 9p21.3 non-codingregion resulted in a severe decrease in cardiac *CDKN2A/B* expression [35], suggesting that the presence of risk alleles in the 9p21.3 region may affect development of CHD through alteration of vascular cell proliferation.

Strengths of the present study include data from large community-based studies, similarity in CAC measurements from the different CT scanners used in the two cohorts, and similarity in imputation strategies and statistical methods. In addition, we utilized strict imputation quality control (only using SNPs with an imputation RSQ ≥ 0.8), which likely decreased the potential for false positive results from poor imputation quality of genotypes. As well, the minor allele frequency of our significant and replicated SNP, rs2069416 (0.37 and 0.33 for GENOA and FHS, respectively), closely matches the reported frequency of 0.36 for a European population in the 1000 Genomes Project data (http://browser.1000genomes.org). Finally, multiple RFs were included in the association and interaction analyses in contrast to most other studies that have included just age and sex [15].

Limitations include the differences in cohort inclusion criteria. The cohorts were entirely of European descent, thus limiting generalizations that can be drawn from these findings. More work is needed to replicate that the 9p21.3 region is involved in gene-BP interactions in other racial and ethnic groups. In addition, this investigation examined a restricted number of SNPs that had first been identified in the CHARGE CAC GWAS [15]. This limited the inferences, as there are likely other loci that contribute to gene-BP interactions in the extent of CAC. Finally, multiple testing is an issue in all genetic association studies and must be accounted for in the results. We have applied Bonferroni correction for the number of SNPs tested to the final meta-analysis results, even though this approach is likely too conservative since the seven SNPs considered here are not completely independent of one another. As well, there is limited statistical power to detect gene-by-environment interactions. Due to these considerations, we used P < 0.05 in both cohorts as a cut-off for declaring significance. One SNP, rs2069416, had significant interactions with DBP under these criteria.

## Conclusions

Our study identified a novel and replicated SNP-by-DBP interaction for rs2069416 that extends our knowledge of the contributions to variation in CAC quantity. This finding may also implicate a genetic pathway through which BP (for which the genetic loci identified for association with BP so far do not overlap with those for coronary atherosclerotic traits) affects CHD pathogenesis. Given the public health importance of morbidity and mortality of CHD, further study of gene-BP interactions will be important as a potential pathway for prevention and therapy.

## Abbreviations

BP: Blood pressure

CAC: Coronary artery calcification

CHARGE: Cohorts for Heart and Aging Research in Genetic Epidemiology

CHD: Coronary heart disease

CT: Computed tomography

DBP: Diastolic blood pressure

EBCT: Electron beam computed tomography

FHS: Framingham Heart Study

GENOA: Genetic Epidemiology Network of Arteriopathy

GWAS: Genome wide association study

HDL: High density lipoprotein

LD: Linkage disequilibrium

LDL: Low density lipoprotein

MDCT: Multi-detector computed tomography

RF: Risk factor

SBP: Systolic blood pressure

SNP: Single nucleotide polymorphism

## Competing interests

The authors declared that they have no competing interests.

## Authors’ contributions

DSK participated in the study design, performed the statistical analysis, and drafted the manuscript; JAS participated in the study design, coordinated the replication of results in FHS, helped draft the manuscript, performed statistical analysis for the manuscript revision and prepared the response to the editor and reviewers; CYW assisted in preparing the genotype data for analysis; LFB participated in the study design and helped draft the manuscript; PAP participated in the study design and helped draft the manuscript; YVS coordinated and prepared data for analysis and helped draft the manuscript; PFSII provided the CAC data for GENOA; STT provided the examination and laboratory data for GENOA; SLRK conceived of the study, participated in the study design, coordinated the analysis, and helped draft the manuscript. All authors read and approved the final manuscript.

## Additional file

## Supplementary Material

Additional file 1: Contains additional details about the measurement and statistical methods used in this study, results from association analyses between the risk factors and CAC quantity and between SNPs and CAC quantity, the linkage disequilibrium plot for GENOA, and a plot of the relationship between rs2069416 genotype, pulse pressure, and CAC quantity. Click here for file
