# Supplementary material for: The relationship between diastolic blood pressure and coronary artery calcification is dependent on single nucleotide polymorphisms on chromosome 9p21.3
Source: BMC Med Genet. 2014 Sep 4;15:89. doi: 10.1186/s12881-014-0089-2 (PMC4168694; doi:10.1186/s12881-014-0089-2)
Supplement: Additional file 1: — Contains additional details about the measurement and statistical methods used in this study, results from association analyses between the risk factors and CAC quantity and between SNPs and CAC quantity, the linkage disequilibrium plot for GENOA, and a plot of the relationship between rs2069416 genotype, pulse pressure, and CAC quantity. [file s12881-014-0089-2-S1.docx]

Additional file

The Relationship between Diastolic Blood Pressure and Coronary Artery Calcification is Dependent on Single Nucleotide Polymorphisms on Chromosome 9p21.3

Kim DS, et al.

**Plotting of Predicted Values of Adjusted ln(CAC score+1)**

Predicted values of ln(CAC score+1), adjusted for covariates as described in the Methods, are defined as the sum of the estimated residual of ln(CAC score +1) plus the mean ln(CAC score+1) (GENOA = 2.88, FHS = 3.15) in each cohort. Plots were constructed using the R statistical language.

First, we classified the imputed genotypes into three categories (0-0.49=0, indicating two non-coding alleles; 0.5-1.49 = 1, indicating a heterozygote genotype; 1.5-2 = 2, indicating two coding alleles). We then plotted the predicted value of adjusted ln(CAC score+1) versus DBP for each genotype category. Finally, we created a line for each categorical genotype’s predicted value of adjusted ln(CAC score+1) using the intercept term along with the beta coefficients for the main effects of the SNP and DBP, as well as the beta coefficient for the SNP-DBP interaction terms.

**Linkage Disequilibrium:**

Linkage disequilibrium (LD), as measured by R^2^, was estimated and plotted using the R-plugin LDheatmap (http://cran.r-project.org/web/packages/LDheatmap/LDheatmap.pdf). To adjust for the GENOA sibship structure, we obtained a subset (N=461) of unrelated participants from our full cohort by randomly selecting one participant from each sibship. We then used this unrelated subset to estimate LD.

**Risk Factor Measures**

**Blood Pressure:**

In the GENOA study, blood pressure measurements were made with a random zero sphygmomanometer at three time points from each participant’s right arm. All measurements were obtained after five minutes of sitting, in accordance with the Joint National Committee guidelines [S1]. In GENOA, the second and third measurements were averaged. In the FHS study, blood pressure measurements were made by a physician with a mercury sphygmomanometer using the participant’s left arm. In FHS, the first and second measurements were averaged. Hypertension status was determined through either: (a) prior diagnosis by a physician and use of current anti-hypertensive medication or (b) average systolic blood pressure (SBP) ≥140mmHg or diastolic blood pressure (DBP) ≥90mmHg.

**Diabetes:**

Fasting glucose levels (mg/dL) were determined from blood samples obtained via venipuncture after an overnight fast. Diabetes status was determined based on past medical history data (past diagnosis or current prescription of anti-diabetes medication) combined with a follow-up laboratory test of fasting blood sugar. For a negative past medical history but a current fasting glucose of ≥126 mg/dL, a determination of positive diabetes status was made for the purposes of study analysis.

**Tobacco Use:**

Pack years, defined as the number of years smoking multiplied by the number of packs smoked per day, were calculated from participants’ medical histories. Pack years was natural log transformed (ln(pack years + 1)) to reduce skewness in both cohorts.

**Lipids:**

Blood levels of total cholesterol, high-density lipoprotein (HDL) cholesterol, and triglycerides were determined via analysis of blood samples drawn after an overnight fast. Low-density lipoprotein (LDL) cholesterol was then calculated using the Friedewald method [S2]. The ratio of LDL to HDL (LDL:HDL) was used in analyses. Use of statin medications was recorded for all participants.

**Figure Legend**

Figure S1. Summary of the pair-wise linkage disequilibrium relationships between the seven SNPs in 9p21.3 studied for gene-by-environment interactions with systolic and diastolic blood pressure.

Figure S2. Relationship between pulse pressure and CAC quantity by rs2069416 genotype in the GENOA Study.

Table S1. Risk factor associations with coronary artery calcification quantity.

| Risk Factor | GENOA Coefficient (SE) | GENOA  P | FHS Coefficient (SE) | FHS  P |
| --- | --- | --- | --- | --- |
| Age, years | 0.107 (0.0076) | <2x10^-16^ | 0.106 (0.0080) | <2x10^-16^ |
| Sex (Women) | -1.201 (0.139) | <2x10^-16^ | -1.775 (0.133) | <2x10^-16^ |
| Hypertensive medication use | 0.455 (0.142) | 1.47 x 10^-3^ | 0.528 (0.147) | 3.55 x 10^-4^ |
| Systolic Blood Pressure, mmHg | 0.0133 (0.0048) | 6.15 x 10^-3^ | 0.0147 (0.0048) | 2.44 x 10^-3^ |
| Diastolic Blood Pressure, mmHg | -0.0154 (0.0086) | 0.0725 | -0.0256 (0.0086) | 3.28 x 10^-3^ |
| ln(Pack Years+1) | 0.221 (0.399) | 4.03 x 10^-8^ | 0.264 (0.0396) | 3.84 x 10^-11^ |
| Diabetes Status | 0.211 (0.270) | 0.435 | -0.215 (0.327) | 0.511 |
| Fasting Glucose, mg/dL | 0.0064 (0.0038) | 0.096 | 0.0108 (0.0043) | 0.011 |
| Statin Use | 0.532 (0.151) | 4.74 x 10^-4^ | 0.905 (0.168) | 9.75 x 10^-8^ |
| LDL:HDL | 0.242 (0.0643) | 1.72 x 10^-4^ | 0.0423 (0.065) | 0.518 |

Table S2. SNP associations with risk factor adjusted coronary artery calcification quantity.

| SNP | GENOA Coefficient | GENOA  P* | FHS  Coefficient | FHS  P* | Position^†^ | Closest Reference Gene^‡^ |
| --- | --- | --- | --- | --- | --- | --- |
| rs3731239 | 0.202 | 0.037 | 0.160 | 0.128 | 21964218 | *CDKN2A* |
| rs1333040 | 0.100 | 0.217 | -0.207 | 0.017 | 22073404 | *CDKN2B-AS1* |
| rs3218009 | -0.103 | 0.357 | 0.166 | 0.222 | 21988757 | *CDKN2B-AS1* |
| rs1333050 | 0.082 | 0.412 | -0.409 | 3.57x10^-4^ | 22115913 | *(CDKN2B-AS1)* |
| rs1333049 | -0.039 | 0.615 | 0.315 | 2.43x10^-4^ | 22115503 | *(CDKN2B-AS1)* |
| rs2069416 | 0.028 | 0.742 | 0.131 | 0.162 | 22000004 | *CDKN2B-AS1* |
| rs2069418 | -0.011 | 0.892 | 0.134 | 0.132 | 21999698 | *CDKN2B-AS1* |

*****Adjusted for: age, sex, systolic blood pressure, diastolic blood pressure, anti-hypertensive medication use, ln(pack years+1), fasting glucose levels, diabetes status, LDL:HDL, and statin drug use.

^†^ Position information from hgRef build 36.3.

^‡^ Genes for SNPs that are outside the transcript boundary of the protein-coding gene are shown in parentheses [e.g., (*CDKN2B-AS1*)].

**Figure S1.**

**
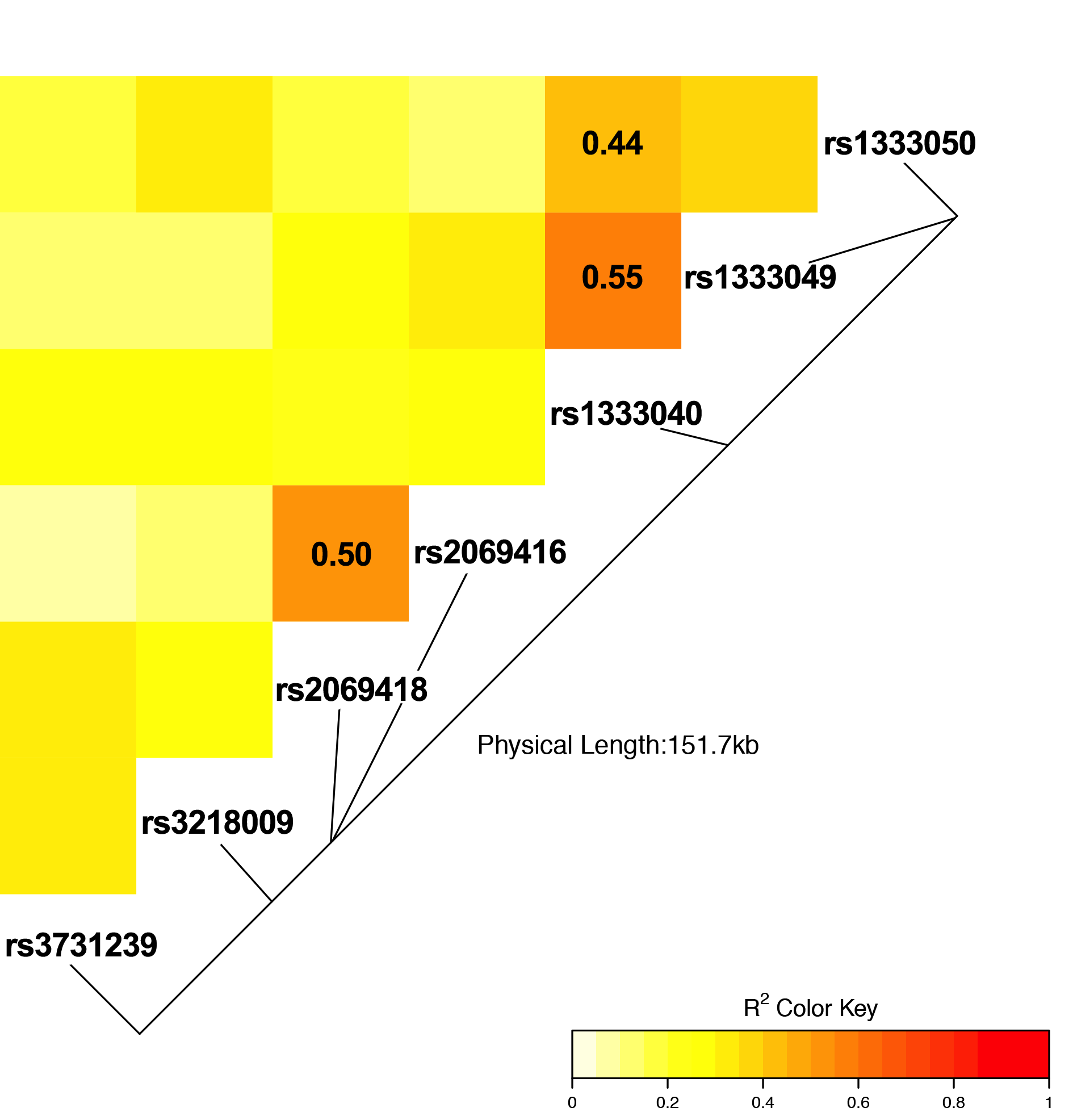
**

**Figure S2.**

**
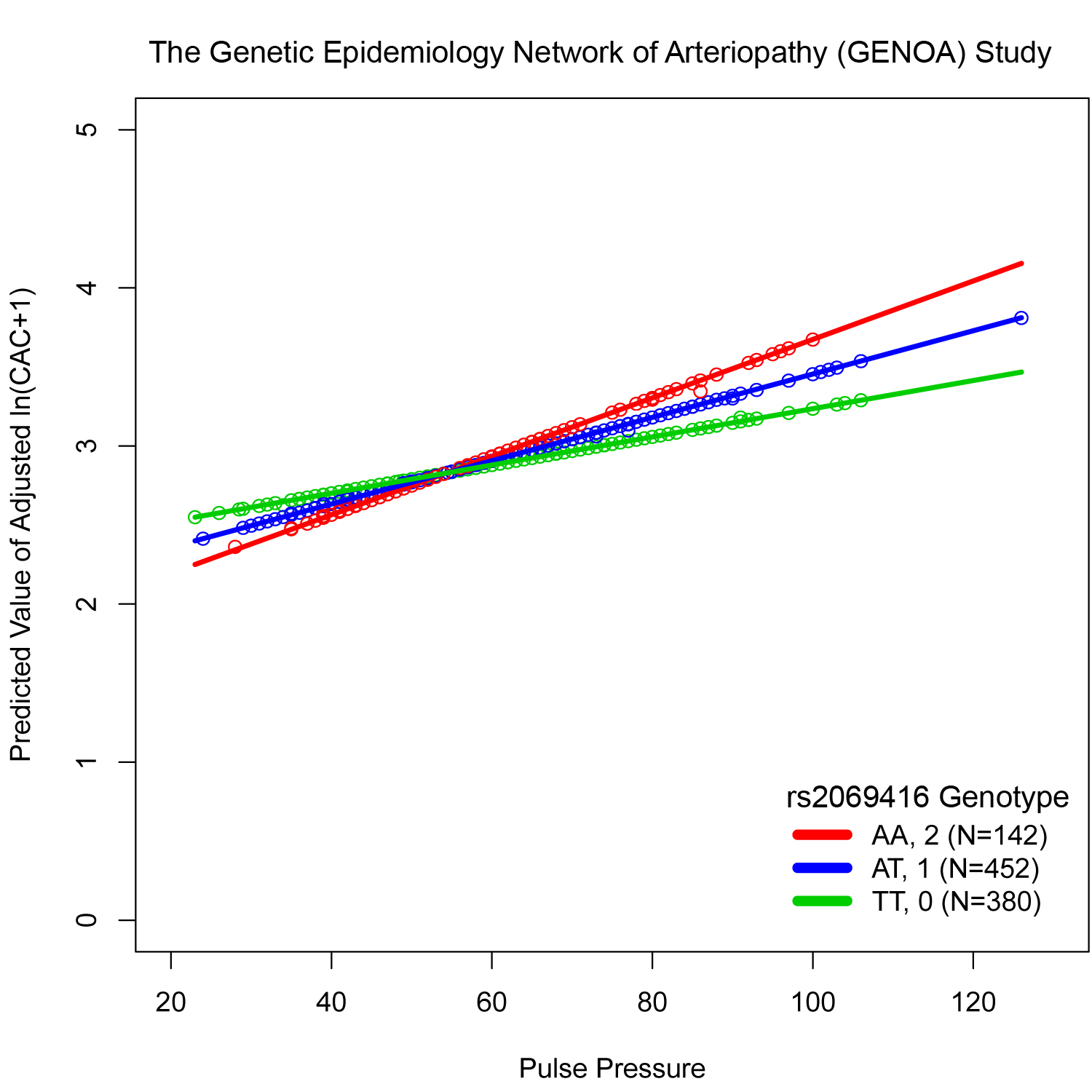
**

**References**

[S1] Chobanian A, Bakris G, Black H, Cushman W, Green L, Izzo J, Jones D, Materson B, Oparil S, Wright J, Roccella E. The Seventh Report of the Joint National Committee on Prevention, Detection, Evaluation, and Treatment of High Blood Pressure: The JNC 7 Report. JAMA 2003, 289:2560-2572.

S[2] Friedewald WT, Levy RI, Fredrickson DS. Estimation of the concentration of low-density lipoprotein cholesterol in plasma, without use of the preparative ultracentrifuge. Clin Chem 1972, 18:499-502.
